# Neurobiological Effects of Transcranial Direct Current Stimulation: A Review

**DOI:** 10.3389/fpsyt.2012.00110

**Published:** 2012-12-28

**Authors:** Liciane Fernandes Medeiros, Izabel Cristina Custodio de Souza, Liliane Pinto Vidor, Andressa de Souza, Alícia Deitos, Magdalena Sarah Volz, Felipe Fregni, Wolnei Caumo, Iraci L. S. Torres

**Affiliations:** ^1^Post-Graduate Program in Biological Sciences, Department of Physiology, Universidade Federal do Rio Grande do SulPorto Alegre, Brazil; ^2^Pharmacology Department, Institute of Basic Health Science, Universidade Federal do Rio Grande do SulPorto Alegre, Brazil; ^3^Laboratory of Pain and Neuromodulation, Hospital de Clínicas de Porto AlegrePorto Alegre, Brazil; ^4^Post-Graduate Program in Medical Sciences, School of Medicine, Universidade Federal do Rio Grande do SulPorto Alegre, Brazil; ^5^Charité – Universitätsmedizin BerlinBerlin, Germany; ^6^Laboratory of Neuromodulation, Department of Physical Medicine and Rehabilitation, Harvard Medical School, Spaulding Rehabilitation Hospital and Massachusetts General HospitalBoston, MA, USA

**Keywords:** tDCS, neurobiology, neuromodulation, functional effects, long-term depression, long-term potentiation

## Abstract

Transcranial Direct Current Stimulation (tDCS) is a non-invasive brain stimulation technique that is affordable and easy to operate compared to other neuromodulation techniques. Anodal stimulation increases cortical excitability, while the cathodal stimulation decreases it. Although tDCS is a promising treatment approach for chronic pain as well as for neuropsychiatric diseases and other neurological disorders, several complex neurobiological mechanisms that are not well understood are involved in its effect. The purpose of this systematic review is to summarize the current knowledge regarding the neurobiological mechanisms involved in the effects of tDCS. The initial search resulted in 171 articles. After applying inclusion and exclusion criteria, we screened 32 full-text articles to extract findings about the neurobiology of tDCS effects including investigation of cortical excitability parameters. Overall, these findings show that tDCS involves a cascade of events at the cellular and molecular levels. Moreover, tDCS is associated with glutamatergic, GABAergic, dopaminergic, serotonergic, and cholinergic activity modulation. Though these studies provide important advancements toward the understanding of mechanisms underlying tDCS effects, further studies are needed to integrate these mechanisms as to optimize clinical development of tDCS.

## Introduction

Transcranial Direct Current Stimulation (tDCS) has been utilized for the modulation of cortical excitability (Nitsche and Paulus, 2000; Fregni et al., 2005; Dieckhöfer et al., 2006; Nitsche et al., 2007; Wagner et al., 2007b) in various diseases, such as depression, chronic pain, stroke, and Parkinson’s disease (Hansen et al., 2010; Lindenberg et al., 2010; Antal and Paulus, 2011; Borckardt et al., 2011, 2012; Riberto et al., 2011; DaSilva et al., 2012; Knotkova et al., 2012; Kumru et al., 2012). tDCS consists of applying direct current (DC) over the scalp using electrodes that are enclosed in perforated sponge pockets soaked with a saline solution or a rubber electrode with conductive gel (Vanneste et al., 2010; DaSilva et al., 2011). It effects depend on the following factors: the size, polarity and position of the electrodes, the applied current intensity, the density and duration of stimulation, and the properties of the tissue in the stimulated area.

This technique can induce long-lasting and polarity-specific changes in the excitability of the motor cortex in humans (Nitsche and Paulus, 2001; Lang et al., 2004). Depending on the current flow, it can increase or decrease neuronal excitability. The mechanisms are electrode-dependent and involve either (1) membrane depolarization (increased spontaneous firing and excitability of the cortical neurons for anodal stimulation) or (2) membrane hyperpolarization (decreased neuronal firing and excitability for cathodal stimulation; Nitsche and Paulus, 2000, 2001; Nitsche et al., 2003a). In the most commonly used procedure, one electrode is placed over a specific site while the other reference electrode is placed over another location to complete the circuit of current flow. The electrode positioning is critical for determining the direction and spatial distribution of the current flow and, ultimately, the effectiveness of the treatment (Utz et al., 2010).

The exact pathways involved in the effects of tDCS are not fully understood (Wagner et al., 2007a; Utz et al., 2010; Stagg and Nitsche, 2011). Thus, more studies to support its clinical application are needed. It is known that weak fields are the basis of the biological effects of tDCS. It is thought that the application of an electric field with sufficient strength and duration can cause a rapid increase in the electrical conductance of biological membranes. This is associated with an increased permeability for ions and small and large molecules. However, the knowledge about the effects on neurotransmission, neurochemical markers, neural pathways, neural tracts, or neural interfaces is incomplete.

Few tDCS studies have been published that assess its underlying neurobiological mechanism. Thus, there is need for further studies to broaden our understanding of the possible neurochemical and neurobiological mechanisms involved. In addition, a better understanding of its possible mechanisms is essential to advance the research and to support its application in a clinical setting. Currently, there are only 123 clinical trials published in English using tDCS. We found 32 studies that assessed the some neurobiological mechanisms.

Neurophysiologists have shown great interest in investigating the effects of low-intensity electrical stimulation, in which the currents used are typically equal to or less than 2 mA, applied to humans (Zaghi et al., 2009). However, studies in human are sometimes insufficient to understand the underlying mechanisms. To address this, we use the translational approach of animal research. The purpose of this review is to summarize the current knowledge and to improve the understanding of the neurobiological mechanisms that may be involved in the effects of tDCS. Moreover, we aim to reveal novel insights into the mechanism of action of the observed clinical responses.

## Methods

This systematic review is based on a literature search using PubMed, Web of Science, OVID MEDLINE, and the Cochrane Library. The keyword “tDCS” was used in combination with other keywords such as “pain,” “chronic pain,” “depression,” “Parkinson,” “stroke,” “cell mechanisms,” “neurobiological mechanisms,” “functional effects,” “intracellular effects,” “receptor,” “long-term depression (LTD),” and “long-term potentiation (LTP).” The term “AND/OR” was used in each combination. The reference sections of the studies that met our inclusion criteria were also manually screened for relevant publications.

### Inclusion criteria

Studies had to meet the following criteria: (1) published in English between 2002 and 2012, (2) report original research, (3) tDCS, (4) the main factors of interest were neurotransmitters, peptides, neurochemical markers, neural pathways, neural tracts, or neural interfaces, and (5) had outcome measures regarding changes in symptoms or electrophysiological or biochemical parameters. Full-text records of each retrieved article were reviewed to determine which studies would be included. We collected information regarding neurobiological mechanisms from human, animal, and cell-culture studies. Moreover, we extracted information on cortical parameters. We systematically screened all articles for the following information: experimental design, sample size, stimulation details (stimulation paradigm and parameters), and main results regarding neurobiological mechanisms. As this review is mainly focused on the neurobiology of tDCS effects, we did not conduct statistical analyses, but instead summarize the results in a narrative format. The exclusion criterion was a lack of original data, such as review articles.

## Results

The final search identified 171 studies. After applying the inclusion and exclusion criteria, we included 32 studies for full-text analysis. We screened the articles according to neurobiological mechanisms, and summarized the results separately for *in vivo* (humans and animals) and *in vitro* studies. Tables 1–4 show the main findings.

**Table 1:** **Neurobiological mechanisms: human *in vivo* studies (*N* = 20)**.

Author (year)	Title	Experiment	*N*	Intervention	Results/insights
Liebetanz et al. (2002)	Pharmacological approach to the mechanisms of transcranial DC-stimulation-induced after-effects of human motor cortex excitability	tDCS 1 mA (5 min) anodal or cathodal over left M1	11 right-handed healthy subjects (8 male)	Carbamazepine (CBZ), dextromethorphan (DMO), or placebo	DMO induced a suppression of the after-effects of both anodal and cathodal stimulation. CBZ eliminated only the anodal effects
Nitsche et al. (2003b)	Pharmacological modulation of cortical excitability shifts. Induced by transcranial direct current stimulation in humans	tDCS 1 mA, cathodal (4 s, 9 min), or anodal tDCS (11–13 min) over left M1	11–14 healthy subjects	Carbamazepine, flunarizine (FLU), dextromethorphan, or placebo	CBZ eliminated only the anodal effects during and after tDCS. FLU has similar effects. The DMO results were similar to those observed in a previous study
Nitsche et al. (2004a)	GABAergic modulation of DC stimulation-induced motor cortex excitability shifts in humans	tDCS 1 mA cathodal (4 s, 5 min, and 9 min) or anodal (11 min) over left M1	6–12 healthy subjects	Lorazepam (LOR) or placebo	LOR promoted a delayed, enhanced, and prolonged increase of excitability induced by anodal tDCS
Nitsche et al. (2004b)	Consolidation of human motor cortical neuroplasticity by d-cycloserine	tDCS 1 mA, cathodal (9 min) or anodal (13 min) over left M1	12 healthy subjects (5 male)	d-cycloserine (d-CYC) or placebo	d-CYC selectively potentiated the duration of increased excitability induced by anodal tDCS
Nitsche et al. (2004c)	Catecholaminergic consolidation of motor cortical neuroplasticity in humans	tDCS 1 mA, cathodal (4 s, 7 min, or 9 min) and anodal (13 min)	5–12 healthy subjects	Amphetaminil (AMP), propanolol (PRO), amphetaminil/dextromethorphan (DMO), or placebo	AMP enhanced and prolonged the increase of the long-lasting excitability changes after anodal tDCS. DMO/AMP blocked any enhancement induced by anodal tDCS. PRO diminished the duration of the after-effects of anodal and cathodal tDCS
Nitsche et al. (2006)	Dopaminergic modulation of long-lasting direct current stimulation of the human motor cortex	tDCS 1 mA, cathodal (9 min), or anodal (13 min) over left M1	4–12 healthy subjects	Sulpiride (SUL), sulpiride/pergolide (PGL), or placebo	SUL almost completely abolished the after-effects of anodal and cathodal tDCS, and promoted a delay in the increase of excitability after anodal tDCS. SUL/PGL did not re-establish the changes induced by tDCS, abolished the delayed excitability increase after anodal tDCS, and prolonged the excitability decrease after cathodal tDCS
Kuo et al. (2007)	Focusing effect of acetylcholine on neuroplasticity in the human motor cortex	tDCS 1 mA, cathodal (9 min), or anodal (13 min) over left M1; followed by iPAS or ePAS	10–12 healthy subjects	Rivastigmine (RIVA) or placebo	RIVA blocked the induction of excitability enhancement after anodal tDCS, showed a tendency to first reduce the inhibition by cathodal tDCS and then later to stabilize the induced inhibition, and enhanced and prolonged the excitability enhancement produced by ePAS and the excitability diminution induced by iPAS
Cheeran et al. (2008)	A common polymorphism in the brain-derived neurotrophic factor gene (BDNF) modulates human cortical plasticity and the response to rTMS	cTBS, iTBS, cathodal tDCS 1 mA (10 min), 1 Hz rTMS, PAS	61 healthy volunteers		Individuals with polymorphism Val66 Met did not present the homeostatic effect expected after pre-conditioning with cathodal tDCS following by 1 Hz rTMS
Kuo et al. (2008)	Boosting focally induced brain plasticity by dopamine	tDCS 1 mA, anodal (13 min), or cathodal (9 min) over left M1	7–11 healthy subjects	Levodopa (l-dopa) or placebo	l-dopa turned the unspecific excitability enhancement caused by anodal tDCS into inhibition, prolonged the excitability diminution induced by cathodal tDCS, and stabilized the PAS-induced synapse-specific excitability increase
Rango et al. (2008)	Myoinositol content in the human brain is modified by transcranial direct current stimulation in a matter of minutes: a ^1^H-MRS study	tDCS 1.5 mA (15 min) anodal or sham over right M1	10 healthy subjects (6 males)		Anodal tDCS increased the myoinositol up to 30 min after stimulation, but only below the electrode
Terney et al. (2008)	Pergolide increases the efficacy of cathodal direct current stimulation to reduce the amplitude of laser-evoked potentials in humans	tDCS cathodal 1 mA (15 min) or control over M1	12 healthy volunteers (5 male)	Pergolide (PGL) or placebo	PGL prolonged the cathodal after-effects, including the reduction of the N2 component for up 2 h and the reduction in pain sensation for up to 40 min
Monte-Silva et al. (2009)	Dose-dependent inverted U-shaped effect of dopamine (D2-like) receptor activation on focal and non-focal plasticity in humans	tDCS 1 mA, anodal (13 min), cathodal (9 min), or sham over left M1; followed by iPAS or ePAS	12 healthy volunteers (6 male)	Ronipirole (RP) or placebo	RP produced an inverted “U” shaped dose-response curve in facilitatory plasticity after tDCS and ePAS and showed the same effect for inhibitory plasticity after tDCS only
Nitsche et al. (2009a)	D1-receptor impact on neuroplasticity in humans	tDCS 1 mA, anodal (13 min), cathodal (9 min) over left M1; followed by iPAS or ePAS	10–12 healthy subjects	Sulpiride (SULP), levodopa (l-dopa)/sulpiride or placebo	SULP abolished the inhibition induced by iPAS, without effecting facilitatory ePAS. l-dopa was able to re-establish the inhibition induced by iPAS
Nitsche et al. (2009b)	Serotonin affects transcranial direct current-induced neuroplasticity in humans	tDCS 1 mA, anodal (13 min), or cathodal (9 min)	12 healthy subjects (8 male)	Citalopram (CIT) or placebo	CIT enhanced and prolonged the facilitation induced by anodal tDCS, whereas it turned cathodal tDCS-induced inhibition into facilitation
Stagg et al. (2009)	Polarity sensitive modulation of cortical neurotransmitters by transcranial stimulation	tDCS 1 mA and anodal, cathodal, or sham (10 min)	7–11 healthy subjects		Anodal tDCS resulted in a local reduction of GABA transmitter, while cathodal tDCS resulted in a decreased glutamate level, which correlated with a reduction in GABA levels
Monte-Silva et al. (2010)	Dosage-dependent non-linear effect of l-dopa on human motor cortex plasticity	tDCS 1 mA, anodal (13 min), or cathodal (9 min) over left M1	12 right-handed healthy subjects (5 men)	Levodopa (l-dopa) or placebo	Low and high doses of l-dopa abolished the facilitatory and inhibitory effects of tDCS
Stagg et al. (2011)	The role of GABA in human motor learning	tDCS 1 mA (10 min) over left M1	12 right-handed healthy subjects (6 males)		A positive correlation was observed between the GABA decrease after anodal tDCS, the degree of motor learning, and the degree of fMRI signal change within the left M1 during learning
Thirugnanasambandam et al. (2011)	Nicotinergic impact on focal and non-focal neuroplasticity induced by non-invasive brain stimulation in non-smoking humans	tDCS 1 mA, anodal (13 min), or cathodal (9 min) over left M1	48 healthy volunteers	Nicotine patch or placebo patch	Nicotine abolished or reduced the inhibitory plasticity after iPAS and cathodal tDCS and the facilitatory plasticity induced by anodal tDCS. The focal facilitatory plasticity (ePAS) was slightly prolonged by nicotine
Antal and Paulus (2011)	A case of refractory orofacial pain treated by transcranial direct current stimulation applied overhand motor area in combination with NMDA agonist drug intake	1st visit: anodal tDCS 1 mA (20 min) for 5 days	1 female patient with persistent orofacial pain	d-cycloserine	The d-cycloserine combined with anodal tDCS resulted in a 60% reduction in pain perception as well as a significant level of pain relief sensation for up to 6 weeks
	2nd visit: cathodal 2 mA (20 min) for 5 days at 2 mA; both over M1	
Chaieb et al. (2012)	Pharmacological modulation of the short-lasting effects of antagonistic direct current stimulation over the human motor cortex	tDCS 1 mA, 10 min (5 min anodal–5 min cathodal or vice-versa) over left M1	8 healthy subjects (6 male)	d-cycloserine (d-CYC), pergolide (PGL), or placebo	The second stimulation produced increases in excitability following anodal stimulation and inhibition following cathodal stimulation. After d-CYC, only inhibition was observed (for both the cathodal-anodal and anodal-cathodal stimulation)

*iPAS, inhibitory paired associative stimulation; ePAS, excitatory paired associative stimulation; cTBS, continuous theta burst; iTBS, intermittent theta burst; rTMS, repetitive magnetic stimulation; PAS, paired associative stimulation; N2 component, negative wave approximately 200 ms as revealed by event-related-potentials measured by electroencephalography*.

### Neurobiological mechanisms

One of the most common ways that we can improve our understanding of neurobiological mechanisms is through pharmacological intervention. Numerous studies have attempted to understand the mechanism of action related to the tDCS neuromodulation technique (Liebetanz et al., 2002; Nitsche et al., 2006; Monte-Silva et al., 2009). It is important to note that these investigations include healthy volunteers as well as patients. In addition, *in vitro* studies and experimental research in animal models can help elucidate the possible mechanisms involved in tDCS.

#### In vivo – humans

A total of 20 articles reported tDCS experiments in humans. The results are presented in Table 1. Most of the articles used pharmacological interventions to characterize the after-effects of tDCS, some of them analyzing the short and long-lasting effects after tDCS. The use of drugs that interact in diverse systems, such as GABAergic, serotoninergic, and cholinergic, can contribute to clarify the some of the neurobiological mechanisms of action related to after-effects of tDCS. The results from these studies demonstrate that a variety of systems can be involved in the mechanisms of action of tDCS. All these articles investigated healthy subjects, except for one case report (Antal and Paulus, 2011).

#### In vitro

A total of six articles reported basic DC experiment. The results are given in Table 2. Studies *in vitro* can bring us the membrane and intracellular mechanisms involved in the effects of DC stimulation. The studies described that the intracellular calcium can be related to one pathway mechanism of tDCS. The BDNF-secretion may be other pathway that can explain the after-effects of DC stimulation.

**Table 2:** **Neurobiological mechanisms: *in vitro* studies (*N* = 6)**.

Author (year)	Title	Experiment	*N*	Model	Results/insights
Khatib et al. (2004)	Physiologic electrical stimulation provokes intracellular calcium increase mediated by phospholipase C activation in human osteoblasts	Electrical stimulation, 2 V/cm	Cells at 60–70% confluence	Osteoblasts cell culture	Electrical stimulation promoted an increase in [Ca^2+^]_i_ that showed a partial inhibition after blocking cation channels or chelating [Ca^2+^]_i_. A phospholipase C inhibitor completely abolished the [Ca^2+^]_i_ increase
Radman et al. (2009)	Role of cortical cell type and morphology in subthreshold and suprathreshold uniform electric field stimulation *in vitro*	DC stimulation, anodal ∼5 mV/mm up to ∼±30 mV/mm	Coronal slices (300 μm) of primary motor cortex (M1) −51 neurons (Pyramidal cells)	Tissue model	The cells responded to DC in a subthreshold and suprathreshold uniform electric field. The importance of the morphology and type of cell in mediating the response to the stimulus was discussed
Fritsch et al. (2010)	Direct stimulation promotes BDNF-dependent synaptic plasticity: potential implication for motor learning	DC stimulation 10 μA	Not described	Coronal mouse slices	They proposed that DCS could induce synaptic plasticity *in vitro* in brain regions that do not respond to conventional protocols. This was dependent on enhanced BDNF-secretion and TrkB-activation
Dubé et al. (2012)	Human keratinocytes respond to direct stimulation by increasing intracellular calcium: preferential response of poorly differentiated cells	Electrical field of 200 mV/mm	The cells were plated into six wells culture dishes) cells/cm^2^ and cultured until 80% confluence was reached	Keratinocytes cell culture	Stimulation induced an increase in intracellular [Ca^2+^]. The extracellular calcium was responsible for this increase, and it was mediated in part by L-type voltage-gated calcium channels. The increase was only detected in involucrin-negative keratinocytes
Ruohonen and Karhu (2012)	tDCS possibly stimulates glial cells	DC stimulation in E-field – 2-mA current for tDCS – 20 mV (2 mA/50 mA) = 0.8 mV	Theoretical analysis	Glial cells	They considered the possibility of glial mechanisms could be modulated by tDCS
Ranieri et al. (2012)	Modulation of LTP at rat hippocampal CA3–CA1 synapses by direct current stimulation	DCS anodal or cathodal, 50 stimuli at 100 Hz (500 ms each) repeated every 20 s	Not described	Hippocampal slices from male Wistar rats	They suggested that tDCS can modulate LTP in intact human brain

*DC, direct current stimulation; [Ca^2+^]_i_, calcium intracellular; BDNF, brain-derived neurotrophic factor; TrkB, tyrosine kinase B; LTP, long-term potentiation*.

#### In vivo – animals

A total of three articles reported DC stimulation experiments in animals. The results are summarized in Table 3. These results demonstrate that DC stimulation can promote neuroprotective or neuroplasticity effects in rat animal models. In addition, it was demonstrated modulation in the learning process after stimulation using rabbits.

**Table 3:** **Neurobiological mechanisms: *in vivo* animals (*N* = 3)**.

Author (year)	Title	Experiment	*N*	Results/insights
Kim et al. (2010)	Functional and histological changes after repeated transcranial direct current stimulation in a stroke model	Anodal or cathodal tDCS, 0.1 mA for 30 min for 2 weeks	41 Sprague-Dawley rats	Anodal stimulation showed a neuroprotective effect (functional improvement and well-preserved white matter axons)
Márquez-Ruiz et al. (2012)	Transcranial direct current stimulation modulates synaptic mechanisms involved in associative learning in behaving rabbits	Anodal or cathodal tDCS from 0.5 to 2 mA (immediate effects) and 1 mA for 20 min (after-effects) over somatosensory (S1) cortex	13 rabbits	Associative learning is modulated by tDCS. Changes were observed in the amplitude and area of the S1 components following anodal or cathodal stimulation. tDCS modulates paired-pulse responses. LTD evoked in the somatosensory cortex after cathodal tDCS is prevented by blocking adenosine A1 receptors
Yoon et al. (2012)	Functional improvement and neuroplastic effects of anodal transcranial direct current stimulation (tDCS) delivered 1 day vs. 1 week after cerebral ischemia in rats	Anodal or sham tDCS, 0.2 mA for 20 min for 5 days	30 male Sprague-Dawley rats	Anodal tDCS modulated neural plasticity around the ischemic penumbra and even in the contralesional area without aggravating the infarction volume or causing metabolic alterations

*LTD, long-term depression*.

### Cortical excitability

We included four articles that associated cortical excitability parameters with neurobiological mechanisms. The parameters of cortical excitability can contribute to a better understanding of the effects of neuromodulatory techniques, such as tDCS. Transcranial magnetic stimulation (TMS) is a tool that can be used for evaluating the parameters of cortical excitability in response to neurostimulatory interventions. The results demonstrated the polarity-specific response of tDCS, anodal stimulation increases MEPs and cathodal decreases it. Most of studies were performed in healthy subjects. The results are given in Table 4.

**Table 4:** **Cortical parameters (*N* = 4)**.

Author (year)	Title	Experiment	*N*	Results/insights
Lang et al. (2004)	Effects of tDCS stimulation over the human motor cortex on corticospinal and transcallosal excitability	tDCS 1 mA anodal or cathodal (10 min) over left M1	8 right-handed healthy subjects (5 male)	Increased or decreased MEPs according to the specific polarity in the left hemisphere. The duration of TC evoked from the right M1 was shortened or prolonged according to the specific polarity
Hasan et al. (2011)	Dysfunctional long-term potentiation-like plasticity in schizophrenia revealed by tDCS	tDCS 1 mA (3 min) anodal over left M1	44 individuals (22 paranoid schizophrenia were compared with 22 matched healthy subjects)	Anodal tDCS resulted in a reduction in LTP-like plasticity in multi-episode schizophrenia patients compared to recent-onset schizophrenia patients and healthy controls. All schizophrenia patients demonstrated reduced cortical inhibition
Polanía et al. (2011)	Introducing graph theory to track for neuroplastic alterations in the resting human brain: a tDCS study	tDCS 1 mA (10 min) anodal or sham over left M1	13 healthy volunteers (6 male)	Anodal tDCS increased the nodal minimum path lengths in the left somatomotor (SM1) cortex, i.e., the number of direct functional connections from the left SM1 to the topologically distant gray matter voxels was significantly decreased. The functional coupling between the premotor and superior parietal areas with the left SM1 was significantly increased. The nodal connectivity degree in the left posterior cingulated cortex area and in the right DLPFC was significantly increased
Scelzo et al. (2011)	Increased short latency afferent inhibition after anodal tDCS	tDCS 1 mA (13 min) anodal over primary motor cortex	12 subjects (4 male)	Anodal tDCS promoted increased short latency afferent inhibition (SAI), which can be related to central cholinergic interneuronal circuits

*M1, primary motor cortex; MEPSs, motor evoked potentials; TC, transcallosal inhibition; LTP, long-term potentiation; DLPFC, dorsolateral prefrontal cortex*.

## Discussion

Overall, we reviewed 32 articles in full-text extracting the main findings of tDCS on neurobiological mechanisms. TDCS effects appear to be multifactorial and capable to induce changes in different systems. Thus, the effects underlying tDCS cannot be simplified to only one mechanism. tDCS induces physiological changes that result in local and distant plastic changes. Some of the tDCS effects seem to be associated with homeostatic effects in a facilitatory and/or inhibitory way.

The studies reviewed in this article demonstrate that the plastic changes induced by tDCS involve regulation of a broad variety of neurotransmitters including dopamine, acetylcholine, and serotonin (Kuo et al., 2007; Monte-Silva et al., 2009; Nitsche et al., 2009b), and also affect a variety of different neuronal membrane channels, such as sodium and calcium. Furthermore the induction of tDCS after-effects is associated with synaptic modulation. The after-effects of anodal and cathodal tDCS are influenced by the potentiation of synaptic glutamatergic receptors (Nitsche et al., 2003b). Furthermore, anodal tDCS is also influenced by GABAergic neurotransmission via interneurons (Nitsche et al., 2004a).

We showed several consistent pharmacological approaches to understand the mechanisms of tDCS (Table 1). The DMO (a NMDA-receptor antagonist) induces suppression of the after-effects of both anodal and cathodal stimulation (Liebetanz et al., 2002), while the CBZ (a sodium use-dependent channel blocker) eliminates only the anodal effects (Liebetanz et al., 2002). Similar effect was observed using flunarizine (a calcium channel blocker) in the study of Nitsche et al. (2003c); however with smaller magnitude of effects as compared with carbamazepine. Lorazepam (a GABAergic agonist) and d-cycloserine (d-CYC, a partial NMDA agonist) selectively potentiate the effects of anodal DC with increased excitability (Nitsche et al., 2004a,b). Propranolol (a non-selective β-adrenergic antagonist) decreases the duration of the after-effects of anodal and cathodal stimulation (Nitsche et al., 2006). These data demonstrate the involvement of multiple neurotransmitter functions in the mechanisms of action of tDCS.

Therefore one important concept when understanding the effects of tDCS is to understand that its initial effect on inducing neuronal depolarization or hyperpolarization(Creutzfeldt et al., 1962; Bindman et al., 1964) results also in lasting effects characterized by LTP and LTD like effects (Hattori et al., 1990; Moriwaki, 1991; Islam et al., 1995; Paulus, 2004). These mechanisms are supported by clinical findings, such as enhanced in the learning and antidepressant effects using tDCS over several weeks (Fregni et al., 2006; Boggio et al., 2008; Loo et al., 2010; Brunoni et al., 2011). Overall, these studies provide valuable insights into the mechanisms of action that tDCS exerts on neuronal tissue (for a review, see Nitsche, 2005).

This systematic review also highlights that the anodal effects are associated with modulation of GABAergic interneurons (Nitsche et al., 2004a; Stagg et al., 2009; Stagg and Nitsche, 2011). This effect is evidenced by the effects of tDCS on short-interval intracortical inhibition and intracortical facilitation (Nitsche et al., 2005; Stagg et al., 2009; Stagg and Nitsche, 2011). Given that GABAergic cortical inhibitory interneurons play a role in the early stage of Alzheimer’s disease (Koliatsos et al., 2006), modulation of these interneurons by tDCS is a potential disease-modifying mechanism. Also, a previous magnetic resonance spectroscopy (MRS) study found that tDCS reduces GABA cortical concentrations and this effect is correlated with impaired glutamatergic neuronal activity (Stagg et al., 2009). These tDCS effects reduce the imbalance between these excitatory and inhibitory neurotransmitter systems. In contrast, carbamazepine selectively eliminated the anodal effects, suggesting that the anodal tDCS require initially depolarization of neuronal membrane potentials (Liebetanz et al., 2002). Liebetanz et al. (2002) provided pharmacological evidence that the induction of the after-effects of tDCS requires a combination of glutamatergic and membrane mechanisms, similar to the induction of established types of short or long-term neuroplasticity.

An important concept when considering the mechanism of TDCS is its association with other interventions such as behavioral and/or pharmacological interventions. The combined application of cathodal/anodal tDCS and d-CYC (a partial agonist of NMDA receptors) during a motor learning task showed that the excitability diminution induced by cathodal tDCS prior to motor learning, or an excitability enhancement induced by anodal tDCS combined with d-CYC, impairs learning performance. Neurophysiologically, a decrease in MEP amplitude was observed (Chaieb et al., 2012). In studies combining tDCS with pharmacological interventions, authors found that application of nicotine patch reduces both inhibitory plasticity after cathodal tDCS and the facilitatory plasticity induced by anodal tDCS (Thirugnanasambandam et al., 2011), while acetylcholine enhances the synapse-specific cortical excitability after anodal tDCS (Kuo et al., 2007). In addition, the inhibitory effect of rivastigmine (a cholinesterase inhibitor) on neuroplasticity induced by anodal tDCS seems contradictory to the results obtained from animal studies in which LTP was facilitated by cholinergic stimulation (Brocher et al., 1992; Abe et al., 1994; Hasselmo and Barkai, 1995; Patil et al., 1998; Kuo et al., 2007). However, these different results might be due to methodological difference between these studies. It is possible that synapses that are globally modified by tDCS are more susceptible to cholinergic suppression of synaptic transmission during plasticity induction.

Other neuropsychotropic drugs showed similar modulation of tDCS-induced plasticity. In fact, TDCS effects are shortened by propranolol following 13 min of anodal and 9 min of cathodal tDCS but does not eliminate those (Nitsche et al., 2004c). Moreover, β-adrenergic receptor stimulation may have an important role for the effects of amphetaminil (a precursor of amphetamine) to increase the consolidation of externally induced excitability enhancements. Similar to results obtained for the β-adrenergic receptor in the hippocampus, it has been also shown that dopamine via the D1-receptor facilitates NMDA-dependent excitability and facilitates NMDA-dependent LTP through Cyclic-adenosine-monophosphate-dependent (cAMP) mechanisms (Otmakhova and Lisman, 1996, 1998; Bailey et al., 2000). Furthermore, it was shown that a single administration of amphetaminil induces prominent and long-term enhancements of cortical dopamine signaling (Vanderschuren et al., 1999). In this way, prolonged dopaminergic activation could stabilize the tDCS-induced NMDA-receptor-dependent excitability enhancements.

Additionally, tDCS promotes changes in brain-derived neurotrophic factor (BDNF; Fritsch et al., 2010). The BDNF promotes the survival of neurons (Lefaucheur, 2008a,b) and is important for cell proliferation (Tessarollo, 1998). Given the results from the study of Cheeran et al. (2008) demonstrating that a common polymorphism in the BDNF gene modulates human cortical plasticity, BDNF could be a marker (and potentially also a pathway) for assessing the effects of tDCS on the nervous system.

Also, new approaches, such as BOLD fMRI, can provide critical information on the mechanisms of tDCS. Furthermore, assessments during the execution of tasks or tDCS stimulation both alone and in combination with other interventions can provide new insights into tDCS effects. Overall, there are many neuropharmacological and neurophysiological methods that can improve our understanding in the neurobiological mechanisms involved in the therapeutic effects of tDCS intervention.

### Limitations in the current knowledge

Although tDCS is one of the most investigated techniques of non-invasive brain stimulation, there are relatively few studies investigating the neurobiological mechanisms associated with the tDCS (Tables 1–4). This article provides information regarding mechanisms of action of tDCS, however most of the mechanistic literature investigated tDCS-related neuroplasticity in the motor cortex. Although motor cortex related data may be of some relevance for treatment of disorders such as chronic pain and motor rehabilitation after stroke where the targeted area is M1, results from experiments in this area are less relevant for other critical targets such as dorsolateral prefrontal cortex. Further research is needed to determine if mechanisms found in studies investigating M1 are also relevant to brain target regions.

Another important issue that has not been adequately addressed is whether the neurophysiological findings can be translated into clinical effects. For instance, whether an increase in excitability induced by anodal tDCS translates into increased motor consolidation. Further larger studies need to address this important question. Finally, it is also important the impact of parameters of stimulation in neuroplasticity – i.e., whether longer periods of stimulation lead to beneficial or harmful effects and also to understand the interaction of tDCS with pharmacological treatment in real clinical practice where patients are taking several medications simultaneously.

## Conclusion and Perspectives

In this review, we discuss the mechanisms of the action of tDCS as to understand neurobiology and cell-signaling pathways associated with tDCS effects. Although initial tDCS studies, showed that its effects are related to the intensity, polarity, and duration of stimulation and the brain region stimulated, it is still not clear the optimal parameters of stimulation especially given the dynamic changes of brain excitability. Recent studies in animal and cell models have suggested that tDCS induces plasticity, neuronal viability, neuronal morphology, modulates synaptic transmission, and biosynthesis of molecules. TDCS induces a cascade of events associated with glutamatergic, GABAergic, dopaminergic, serotonergic, and cholinergic activity modulation. In addition, we also show the importance of conducting both experimental and clinical studies to understand tDCS-induced neuroplasticity. Overall, compelling evidence from studies reviewed in this article emphasizes possible approaches to understand the neurobiology of tDCS mechanisms. Additionally, it opens new possibilities for future tDCS research in basic and clinical neuroscience.

## Conflict of Interest Statement

The authors declare that the research was conducted in the absence of any commercial or financial relationships that could be construed as a potential conflict of interest.
